# GB1a Ameliorates Ulcerative Colitis via Regulation of the NF-κB and Nrf2 Signaling Pathways in an Experimental Model

**DOI:** 10.3389/fmed.2021.654867

**Published:** 2021-09-07

**Authors:** Yuanyuan Yu, Congmin Zheng, Xu Lu, Changsheng Deng, Qin Xu, Wenfeng Guo, Qingye Wu, Qi Wang, Changhui Liu, Xinan Huang, Jianping Song

**Affiliations:** ^1^Artemisinin Research Center, Guangzhou University of Chinese Medicine, Guangzhou, China; ^2^The First Affiliated Hospital and The First Clinical Medical School, Guangzhou University of Chinese Medicine, Guangzhou, China; ^3^School of Pharmaceutical Sciences, Guangzhou University of Chinese Medicine, Guangzhou, China

**Keywords:** ulcerative colitis, GB1a, inflammation, oxidative stress, colonic epithelial barrier

## Abstract

Ulcerative colitis (UC) is an inflammatory bowel disease. The intake of African Garcinia Kola nuts has been reported as a therapy for diarrhea and dysentery in the African population. However, the mechanism of action through which Garcinia Kola nuts act to ameliorates UC remains unknown. GB1a is the main active component of Garcinia Kola nuts. In this study, we explored the therapeutic effects and underlying mechanism of GB1a on dextran sodium sulfate (DSS)-induced UC. Human Colonic Epithelial Cells (HCoEpic) were challenged with TNF-α to test the effects of GB1a in protecting against oxidative stress and inflammation *in vitro*. Our data showed that GB1a significantly attenuated DSS-induced colonic inflammatory injury manifested as reversed loss of body weight and disease activity index (DAI) scores in UC mice. We also showed that GB1a improved the permeability of the intestinal epithelium by modulating the expression of tight junction proteins (ZO-1, Occludin). Mechanistically, GB1a may activate the Nrf2 antioxidant signaling pathway and suppress the nuclear translocation of NF-κB in reduced oxidative stress and expression of inflammatory genes induced by TNF-α in HCoEpic cells. Our study suggests that GB1a alleviates inflammation, oxidative stress and the permeability of the colonic epithelial mucosa in UC mice via the repression of NF-κB and activation of Nrf2 signaling pathway.

## Graphical Abstract

GB1a attenuates DSS-induced ulcerative colitis via remodeling of the NF-κB and Nrf2 signaling pathways. 
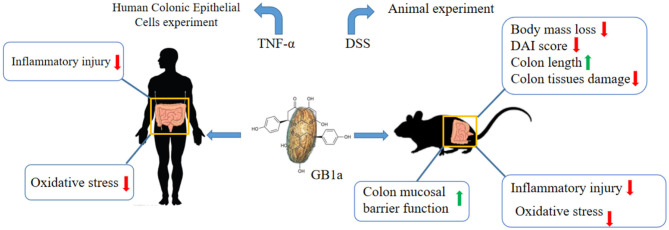

## Introduction

Ulcerative colitis (UC), also known as non-specific ulcerative colitis, is a type of inflammatory bowel disease (IBD) that occurs in the rectum and colon (1). During the development of UC, many pathological lesions occur such as ulcers, crypt abscesses, small vessel inflammation, and reduced numbers of goblet and inflammatory cells (2). Inflammation and ulcerative lesions of the mucosa and submucosa are the main pathological features of UC (3). The main clinical symptoms of the disease include abdominal pain, bloody diarrhea, constipation, and fatigue. These symptoms have a major impact on the quality of life for patients and increase the risk of secondary infections and colon cancer in patients with long-term recurrence (4, 5).

The etiology of UC remains to be fully elucidated but is known to involve interactions between environmental, genetic, and immune factors leading to uncontrolled abnormal immune responses in the intestinal mucosa (6, 7). Studies have identified multiple molecular pathways that are involved in the pathogenesis of UC including the NF-κB pathway, oxidative stress, and the release of related inflammatory cytokines and pro-inflammatory mediators (8–10). Oxidative stress responses result in the infiltration of macrophages into the colon tissues of patients with UC leading to the production of high levels of reactive oxygen species (ROS) (11, 12). These changes act to increase the permeability of the intestinal epithelium and induce further damage in colon tissues leading to the development of intestinal inflammation (13).

Nuclear factor erythroid 2-related factors 2 (Nrf2) is a redox-sensitive transcription factor that protects cells from inflammation and oxidative stress by regulating the transcription of anti-oxidation and detoxification genes including glutathione S-transferase (GST), glutathione peroxidase (GPx), heme oxygenase-1 (HO-1), superoxide dismutase (SOD). Nrf2 enhances the ability of cells to remove electrophilic and reactive oxygen species (ROS) (14–16). Previous studies have shown Nrf2 knockout results in more severe damage in the colon of a UC mouse model which may be to excess generation of ROS generation and inflammatory cytokines (17, 18). Genetic or pharmacological activation of Nrf2 effectively protects mice against the DSS-induced symptoms of UC in mice via remodeling of the Nrf2/ARE and Nrf2/HO-1 pathways.

Previous studies have shown that abnormal activation of NF-κB plays a central role in regulating the release of cytokines in UC patients resulting in severe inflammation and immune response (19, 20). NF-κB and its inhibitor, IκB, stably bind in the cytoplasm. NF-κB dimers are released after degradation of the IκB protein as IκB kinase activation is stimulated by various extracellular factors. Subsequently, NF-κB is further activated by various post-translational modifications and combines with the promoter regions of target genes allowing the expression of downstream targets including TNF-α, IL-6, and IL-1β (21–25). Therefore, the effective suppression of NF-κB may provide a potential therapeutic approach in UC.

GB1a is a bioflavonoid that is extracted from the Garcinia Kola nuts, a tropical evergreen plant of the Garciniaceae and genus Garcinia. Garcinia Kola is widely used as an antioxidant, antibacterial, antiviral, antiulcer, and anti-inflammatory agent (26, 27). Previous studies have reported that flavonoids extracted from Garcinia Kola can reduce inflammation and increase antioxidant capacity by activating Nrf2, yet the active ingredients in the extract remain to be identified (28). In this study, we found that GB1a is effective on DSS-induced UC mice manifested by the recovery bodyweight and decreased DAI scores as well as the improvements in levels of damage in colon tissues. In this study, we explored the therapeutic effects and mechanism of GB1a on dextran sodium sulfate (DSS)-induced UC symptoms.

## Methods and Materials

### Extract Preparation

Garcinia Kola nuts were obtained from Nigeria, Africa. Garcinia Kola nuts were cleaned using fresh tap water to remove dust, air-dried, and then crushed. Extraction was performed twice using 95% (v/v) ethanol and then the solution was evaporated to semi-dryness using a rotary vacuum evaporator at 45°C. The filter residue was added to pure water and refluxed for extraction twice for 1 h. The filtrate was combined and concentrated under reduced pressure to obtain the extract. All of the obtained extracts were dissolved in an appropriate amount of water for extraction and extracted three times with petroleum ether reagent to obtain a petroleum ether layer and a water layer. The water layer was extracted three times with n-butanol to obtain an n-butanol layer and a water layer. The n-butanol layer was concentrated under reduced pressure to obtain the extract.

For High-Performance Liquid Chromatography (HPLC) analysis, 1 mg of extract powder was dissolved in 1 ml of methanol and filtered through a 0.22 μm filter before HPLC analysis. An Agilent 1260 HPLC (Agilent Technologies, Santa, Clara, CA, USA) equipped with a Zorbax Eclipse Plus C_18_ column (ZORBAX SB-C_18_, 9.4 ×250 mm, 5 μm) was used for HPLC analysis and preparation. Chromatographic separation was performed at 30°C with a flow rate of 2.5 mL/min. The injection volume was 50 μL and the ultraviolet detection wavelength was set at 360 nm. The mobile phase consisted of methanol (A) and water (B). The gradient elution conditions of the mobile phase A were: 0–20 min, 53–65%; 20–21 min, 65%; 21–30 min, 65–70%; 30–31 min, 70%; 31–35 min, 70–53%; 35–40 min, 53%. After purification by HPLC, a single compound with a purity of 99.7% in the n-butanol extract layer was obtained (Supplementary Figure 1A). The identification and analysis of the hydrogen (^1^H NMR) and carbon spectra (^13^C NMR) showed that the compound was GB1a (Supplementary Table 1 and Supplementary Figures 1B,C) (29).

### Cell Protocols

Human Colonic Epithelial Cells (HCoEpic) were seeded in 96 well plates with 6 well replicates. After culturing for 24 h, the culture was changed to a medium containing GB1a (drug concentration gradient: 0, 2.5, 5.0, 10, 15, 20, 50, 100, 200, 400 μM) and the cells incubated for 24 h after administration. The culture medium was then aspirated and the cells were incubated with a pre-mixed medium containing CCK-8 (100 μL 1640 medium, 10 μL CCK-8 solution). The OD value was measured at 450 nm using a microplate reader. HCoEpic cells were harvested after incubation for 24 h with 30 ng·ml^−1^ TNF-α (TNF-α model group) or TNF-α plus 20 μM/40 μM GB1a (TNF-a+GB1a group). All experiments were performed in triplicate.

### Animals

Male C57BL/6 mice (6–8 weeks old, 18–20 g) were purchased from the Beijing Weitong Lihua Experimental Animal Technology Co., Ltd. Mice were maintained in a 12-h dark/light cycle environment with a room temperature of 23 ± 2°C and a relative humidity of 55 ± 5%. Mice had free access to a standard diet and purified water. All animal experiments were performed following protocols and guidelines approved by the Animal Ethics Committee of Guangzhou University of Chinese Medicine. All surgeries were performed under sodium pentobarbital anesthesia.

### Induction of Colitis and Treatment Protocol

After 1 week of adaptive feeding, C57BL/6 mice were randomly divided into six groups (7 mice/group) as follows; Control, DSS, DSS+sulfasalazine (SASP, 300 mg·kg^−1^), DSS+GB1a100, DSS+GB1a50, DSS+GB1a25 (GB1a, 100 mg·kg^−1^, 50 mg·kg^−1^, 25 mg·kg^−1^). During the experimental periods, animals received a daily gavage of SASP (300 mg·kg^−1^) or GB1a (25, 50, or 100 mg·kg^−1^) in 0.5% carboxymethyl cellulose from day 1 to day 9. From day 3, for 2 h after administration of GB1a, mice were given 4% DSS (w/v) solution dissolved in sterile distilled water *ad libitum* for 6 days. GB1a and SASP administration continued until the end of the DSS treatment period.

### Evaluation of Colitis

Daily observations were performed to assess the symptoms of colitis (body mass loss, the severity of diarrhea, rectal bleeding). The disease activity index (DAI) was evaluated as described (DAI = Score Weight loss (%) + Stool consistency + rectal bleeding) (30). At the end of the experiment, mice were anesthetized by i.p. administration of 10% chloral hydrate. The entire colon was excised and measured. Portions of the colon were fixed in 4% paraformaldehyde, embedded in paraffin and processed for routine hematoxylin and eosin (H&E) staining for examination under a light microscope. The histological scores of the H&E-stained colon specimens were blindly assessed by two pathologists. Histological sections were scored using a validated scoring system as previously described by Dou et al. (31). The remaining parts of the colons were stored at −80°C for further analysis.

### Determination of Myeloperoxidase (MPO) Activity in Colon Tissues

Inflammation was assessed by measuring tissue myeloperoxidase (MPO) activity that is linearly related to neutrophil infiltration. MPO activity in the supernatant of the colon homogenate of mice was determined using an MPO assay kit according to the manufacturer's instructions (Nanjing jiancheng, Nanjing, China). The values were expressed as units per gram of tissue in each sample and calculated from the following formula: Myeloperoxidase (MPO) Activity (U/g) = (Measure OD value-control OD value)/11.3 × sampling volume (g).

### Assessment of Serum Levels and Antioxidant Parameters

The serum levels of TNF-α and IL-6 were measured using ELISA assay kits (Abclonal Biotechnology Co., Ltd, Wuhan, China) according to the manufacturer's instructions. Assay kits (Nanjing jiancheng, Nanjing, China) were used to measure levels of malondialdehyde (MDA), glutathione (GSH) and superoxide dismutase (SOD) in serum.

### Mitochondrial DNA Copy Number

The mtDNA copy number was used as a marker for mitochondrial density using qPCR as previously reported (32, 33). Briefly, total DNA was isolated from HCoEpic cells using a Universal Genomic DNA Extraction kit (Tiangen, Beijing, China) according to the manufacturer's instructions. The mitochondrial DNA copy number was calculated from the ratio of the mitochondrial-encoded gene COXII and the nuclear-encoded gene GAPDH. The primer sequences of the genes are shown in Supplementary Table 2.

### Western Blotting Analysis

Protein was extracted from HCoEpic cells or mouse colon tissues samples. Equal concentrations of proteins were separated on a 10% SDS-polyacrylamide gel and transferred to polyvinylidenefluoride (PVDF) membranes. Western blotting was performed using specific antibodies (Anti-Nrf2, anti-HO-1, anti-NF-κBp65, anti-ZO-1, anti-Occludin, anti-β-actin, and anti-LaminB) purchased from ABclonal (ABclonal, Biotechnology Co., Ltd.).

### Measurement of ROS

HCoEpic cells were seeded in 6-well plates and treated as previously described. Cells were then incubated with DCFH-DA (5 uM) at 37°C for 0.5 h in the dark. Cells were washed three times with PBS and the fluorescence emission was detected using a fluorescence microscope.

### Management of Fluorescein Isothiocyanate (FITC)-Dextran

At the end of the DSS treatment period, mice were given fluorescein isothiocyanate (FITC)-dextran solution (4 kDa, 600 mg/kg) by oral gavage. Blood samples were collected from the retinal vein after 4 h.

### Statistical Analysis

All results presented in the figures are expressed as the mean ± SEM. The significant differences between multiple groups were detected using a one-way analysis of variance (ANOVA) followed by the least significant difference (LSD) or a Dunnett's test (SPSS20.0). Data were evaluated using GraphPad Prism Version 7.0. *P*-values of < 0.05 were considered statistically significant.

## Results

### GB1a Exerts Anti-inflammatory Effects by Inhibiting the Nuclear Translocation of NF-κB *invitro*

**Figure 1:**
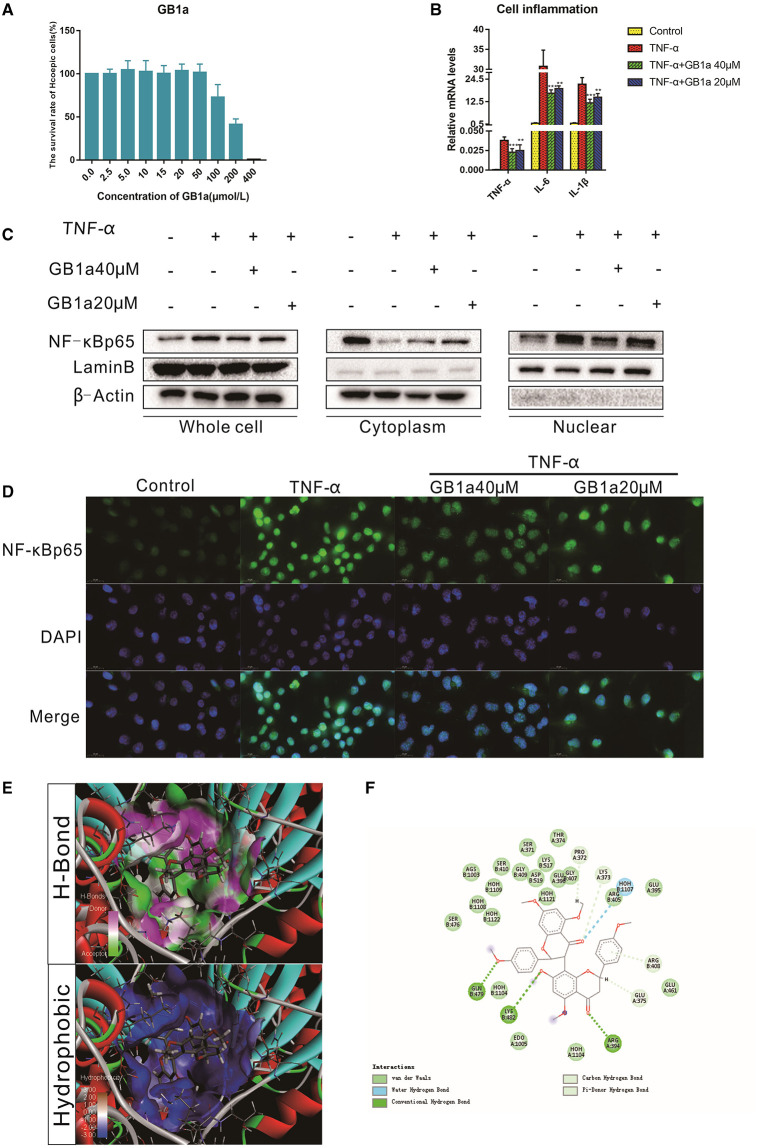
The anti-inflammatory effects of GB1a are mediated by inhibition of NF-κB nuclear translocation *in vitro* **(A)** CCK8 analysis showed the cytotoxicity of GB1a on HCoEpic at different doses. **(B)** GB1a administration reduced the expression of intracellular pro-inflammatory cytokines (TNF-α, IL-6 and IL-1β) in TNF-α-incubated HCoEpic in a dose-dependent manner. **(C)** GB1a treatment could inhibit NF-κB p65 protein expression in the nucleus and block NF-κB p65 translocation to the nucleus. **(D)** Immunofluorescence analysis of NF-κB (green) in HCoEpic. DAPI was used for nuclear staining (blue). **(E)** The 2D structure of the predicted binding of GB1a to NF-κB. **(F)** The molecular docking model of GB1a and NF-κB. Data are presented as means ± SD (*n* = 5/group). **p < 0.01 and ***p < 0.001.vs the TNF-α-incubated group.

To investigate the anti-inflammatory function of GB1a, TNFα-incubated HCoEpic cells were used as previously described (34). Consistent with our hypothesis, GB1a displayed lower cytotoxicity (Figure 1A) and GB1a treatment effectively reduced the expression of pro-inflammatory genes including TNF-α, IL-1β, and IL-6 (Figure 1B). NF-κB is a the master regulator of proinflammatory gene expression. We showed that GB1a could reverse the TNF-α-induced the elevation of NF-κB p65 expression in HCoEpic cells in a dose-dependent manner by inhibiting the nuclear translocation of NF-κB induced by TNF-α (Figures 1C,D). To further explore the underlying mechanism, we performed docking analysis between GB1a and NF-κB. Our molecular docking data indicated a high binding affinity via hydrophilic interactions suggesting a potential role of GB1a in regulating NF-κB activity (Figures 1E,F). To confirm the mechanism is through the NF-κB signal pathway, we transfected the siRNA- NF-κBp65 on HCoEpi cells. As shown in Supplementary Figure 2, the expression of NF-κBp65 was significantly decreased after knocking down NF-κBp65 when compared with control group. In addition, knocking down NF-κBp65 significantly reduced the mRNA levels of TNF-α, IL-1β, and IL-6. As expected, GB1a significantly decreased the mRNA levels of NF-κB p65 and its downstream genes in HCoEpi cells. Altogether, these results demonstrated that GB1a reduced the expression level of NF-κBp65 in HCoEpi. Collectively, these data suggested a potential anti-inflammatory role of GB1a.

### GB1a Activates the Nrf2 Pathway and Alleviate TNF-α-Induced Mitochondrial Injury *in vitro*

**Figure 2:**
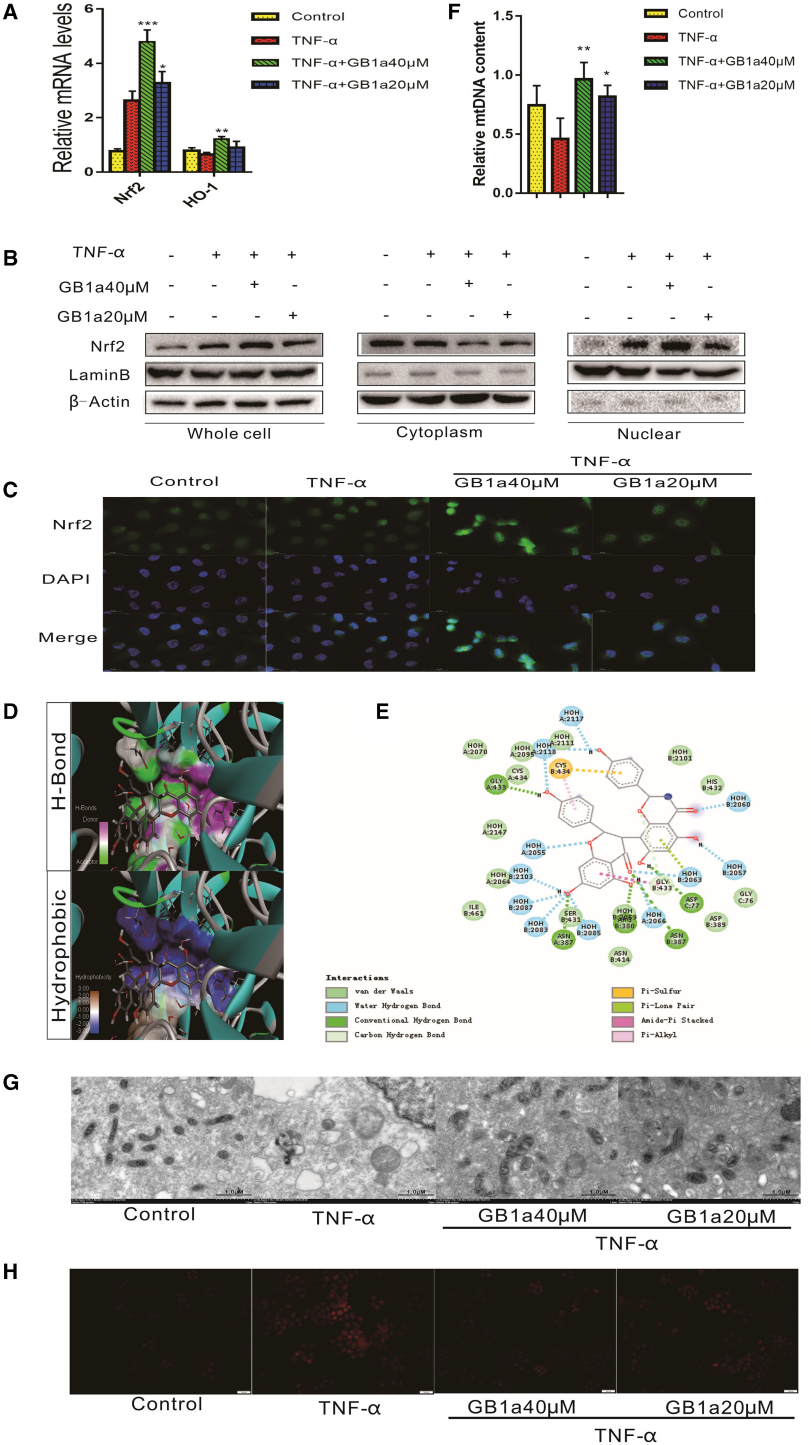
GB1a activates the Nrf2 pathway and alleviates TNF-α-induced mitochondrial injury *in vitro*. **(A)** GB1a treatment upregulated the expression of Nrf2 and HO-1. **(B)** Western blotting results showed that GB1a advances Nrf2 translocation to the nucleus and promotes Nrf2 protein expression in the nucleus. **(C)** Immunofluorescence analysis of Nrf2 (green) in HCoEpic. DAPI was used for nuclear staining (blue). **(D)** The 2D structure of the predicted binding of GB1a to Nrf2. **(E)** The molecular docking model of GB1a and Nrf2. **(F,G)** GB1a treatment improved mitochondrial biogenesis **(F)** and morphology **(G)** in HCoEpic. **(H)** GB1a reduced the levels of ROS in TNF-α-incubated HCoEpic. Data are presented as means ± SD (*n* = 5/group). *p < 0.05, **p < 0.01, and ***p < 0.001 vs. the TNF-α-incubated group.

Previous studies (35, 36) have shown that long-term inflammation results in impaired mitochondrial function and the overproduction of ROS that drives the pathogenesis of UC damage. These data suggest that the suppression of chronic inflammation-induced colonic oxidative stress may colitis. We investigated the effects of GB1a on TNF-α-induced mitochondrial stress and intracellular redox status. GB1a treatment significantly upregulated genes involved in antioxidant pathways including Nrf2 and HO-1 (Figure 2A). In parallel to the enhanced mRNA levels, GB1a significantly promoted Nrf2 protein expression and nuclear translocation (Figures 2B,C). Molecular docking studies revealed that GB1a could bind to the inside of the Nrf2 domain based on the hydrogen, hydrophobic interactions and van der Waals forces (Figures 2D,E). Moreover, GB1a treatment effectively reversed TNF-α-induced mitochondrial loss in HCoEpic cells supported by improved mitochondrial biogenesis and ultrastructural features (Figures 2F,G). These changes led to the attenuation of redox imbalance supported by decreased ROS levels (Figure 2H). Collectively, our results showed that GB1a can directly interact with Nrf2 to facilitate the recruitment of coactivators suggesting that GB1a serves as an Nrf2 agonist.

### GB1a Alleviates the Symptoms of DSS-Induced UC in Mice

**Figure 3:**
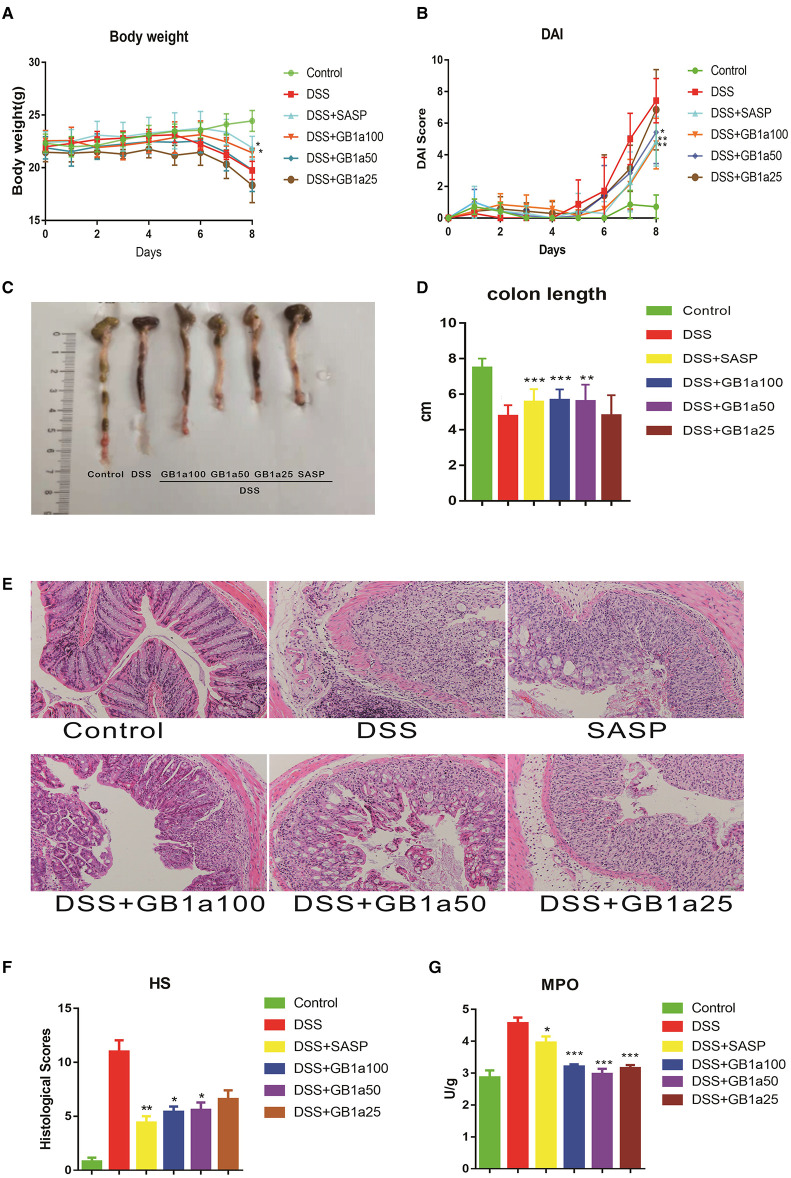
GB1a alleviates the symptoms of DSS-induced UC in mice. **(A–D)** GB1a treatment ameliorated DSS-induced body weight loss **(A)**, DAI scores raising **(B)**, colon shortening **(C,D)**. **(E,F)** GB1a attenuates colonic pathological damage in UC mice **(E)** and decreased the histological scores of the colon tissues **(F)**. **(G)** GB1a intervention significantly reduced the serum MPO activity in UC mice. Data are presented as means ± SD (*n* = 7/group). *p < 0.05, **p < 0.01, and ***p < 0.001 vs. the DSS-incubated group.

Administration of GB1a results in a dose-dependent decrease in body mass compared to DSS-treated mice with concomitant improvements in the DAI score at a higher dose of GB1a (Figures 3A,B). DSS treatment caused significant shortening of the colon compared to the control group which was attenuated in a dose-dependent manner by GB1a post-treatment (Figures 3C,D). Histopathological examination of the mouse colon tissues showed that GB1a treatment effectively reversed the DSS-induced damage in a dosage dependent manner. These findings were supported by observations of a repaired mucosal structure, increased crypt numbers, and reduced inflammatory cell infiltration in the mucosa and submucosa (Figures 3E,F). Furthermore, the MPO activity assay results showed that GB1a intervention significantly reduced MPO activity in the colon tissues of UC mice (Figure 3G). In conclusion, these data indicated that GB1a has potential effects on the treatment of UC.

### The Inhibitory Effects of GB1a Are Dependent on the NF-κB Signaling Pathway in UC Mice

**Figure 4:**
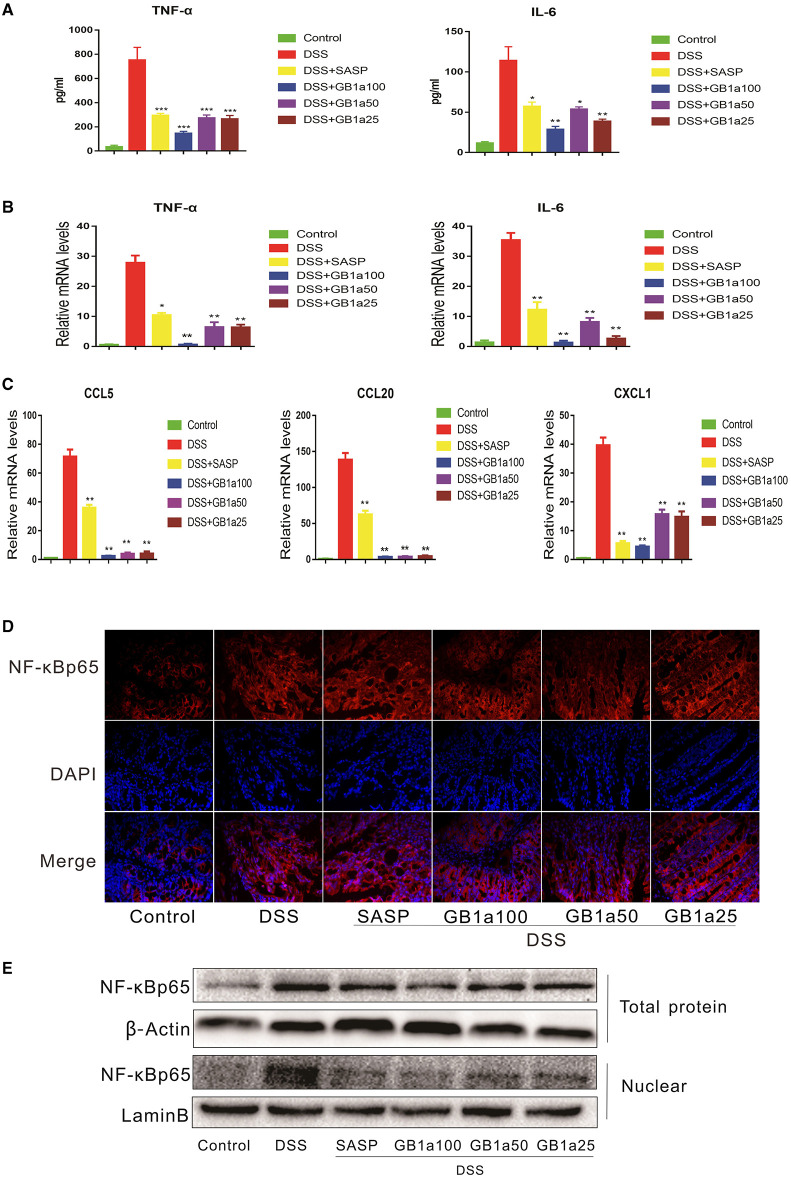
The inhibitory effects of GB1a are dependent on the NF-κB signaling pathway in UC mice. **(A)** Treatment with GB1a down-regulated serum TNF-α and IL-6 levels in UC mice. **(B,C)** GB1a inhibited the expression of TNF-α, IL-6, CCL5, CCL20, CXCL1 in the colon tissue of UC mice. **(D)** Immunofluorescence analysis of NF-κBp65 (red) in the colon of mice. DAPI was used for nuclear staining (blue). **(E)** NF-κBp65 levels in whole and nuclear protein were analyzed by western blotting. Data are presented as means ± SD (*n* = 7/group). *p < 0.05, **p < 0.01, and ***p < 0.001 vs. the DSS-incubated group.

Considering the anti-inflammatory effects of GB1a are mediated by inhibiting the activation of the NF-κB pathway *in vitro*, we hypothesized that GB1a also alleviates DSS-induced UC inflammatory damage through repression of the NF-κB pathway. As predicted, GB1a treatment reversed the DSS-induced increase of pro-inflammatory cytokines including TNF-α and IL-6 (Figure 4A). Also, GB1a treatment inhibited the DSS-induced elevation of IL-6 and TNF-α mRNA expression and inhibited the expression of chemokines including CCL5, CCL20, CXCL1 (Figures 4B,C). Finally, we measured the expression levels of NF-κBp65 in colon tissues by immunofluorescence and western blotting. Our results showed that GB1a inhibited NF-κBp65 expression and blocked NF-κBp65 translocation to the nucleus in DSS mice. These changes led to the amelioration of DSS-induced inflammation in the colon by inhibiting activation of the NF-κB pathway (Figures 4D,E).

### The Effect of GB1a Activation on the Nrf2 Signaling Pathway in UC Mice

**Figure 5:**
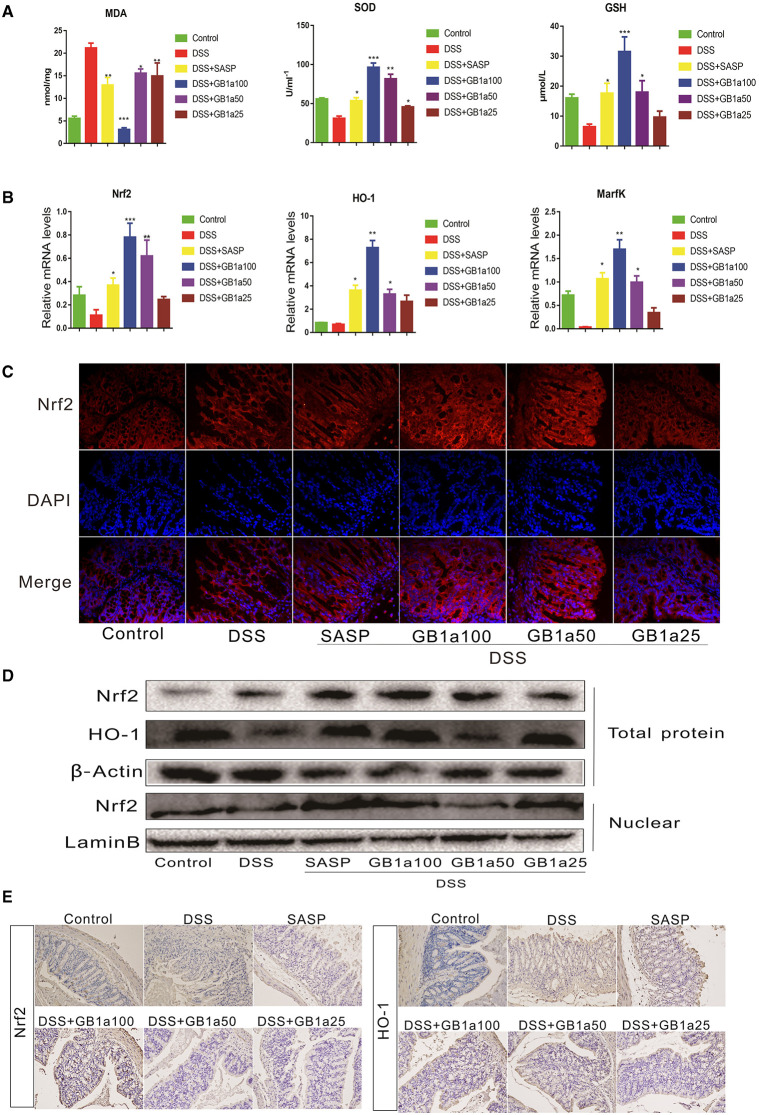
The effects of GB1a on the activation of the Nrf2 signaling pathway in UC mice. **(A)** GB1a administration reduced the levels of oxidants (MDA) and increased the levels of antioxidants (GSH, SOD) in the serum of UC mice. **(B)** GB1a treatment upregulated the expression of antioxidant genes Nrf2, HO-1, and MarfK. **(C)** Immunofluorescence analysis of Nrf2 (red) in the colon of mice. DAPI was used for nuclear staining (blue). **(D)** GB1a administration increased the expression of Nrf2 and HO-1 proteins and promoted Nrf2 translocation to the nucleus. **(E)** Representative images of immunohistochemical staining of Nrf2 and HO-1 in mice colon sections. Data are presented as means ± SD (*n* = 7/group). *p < 0.05, **p < 0.01, and ***p < 0.001 vs. the DSS-incubated group.

Based on the results of our *in vitro* experiments, we further investigated the dependency of the therapeutic effects of GB1a in UC mice via the activation of colonic Nrf2 pathways. We determined the levels of oxidants (MDA) and antioxidants (GSH, SOD) in the serum of mice and the mRNA expression of antioxidant genes (Nrf2, HO-1 and MarfK) in colon tissues. GB1a treatment significantly decreased the levels of the oxidant MDA and increased the level of the antioxidants GSH and SOD (Figure 5A). We also found the upregulated expression of the antioxidant genes, Nrf2, HO-1 and MarfK (Figure 5B). Immunofluorescence staining and western blotting analysis showed that GB1a administration significantly increased Nrf2 expression in colon tissues and promoted Nrf2 translocation to the nucleus (Figures 5C,D). Consistent with the activation of Nrf2 signaling, immunohistochemistry results showed that GB1a treatment significantly enhanced Nrf2 and HO-1 protein expression in inflamed colons compared to mice treated with DSS alone (Figure 5E). Taken together, these results suggest that GB1a exerts an anti-oxidant effect through the Nrf2 signaling pathway to improve the symptoms of UC.

### The Protective Effect of GB1a on the Intestinal Mucosa

**Figure 6:**
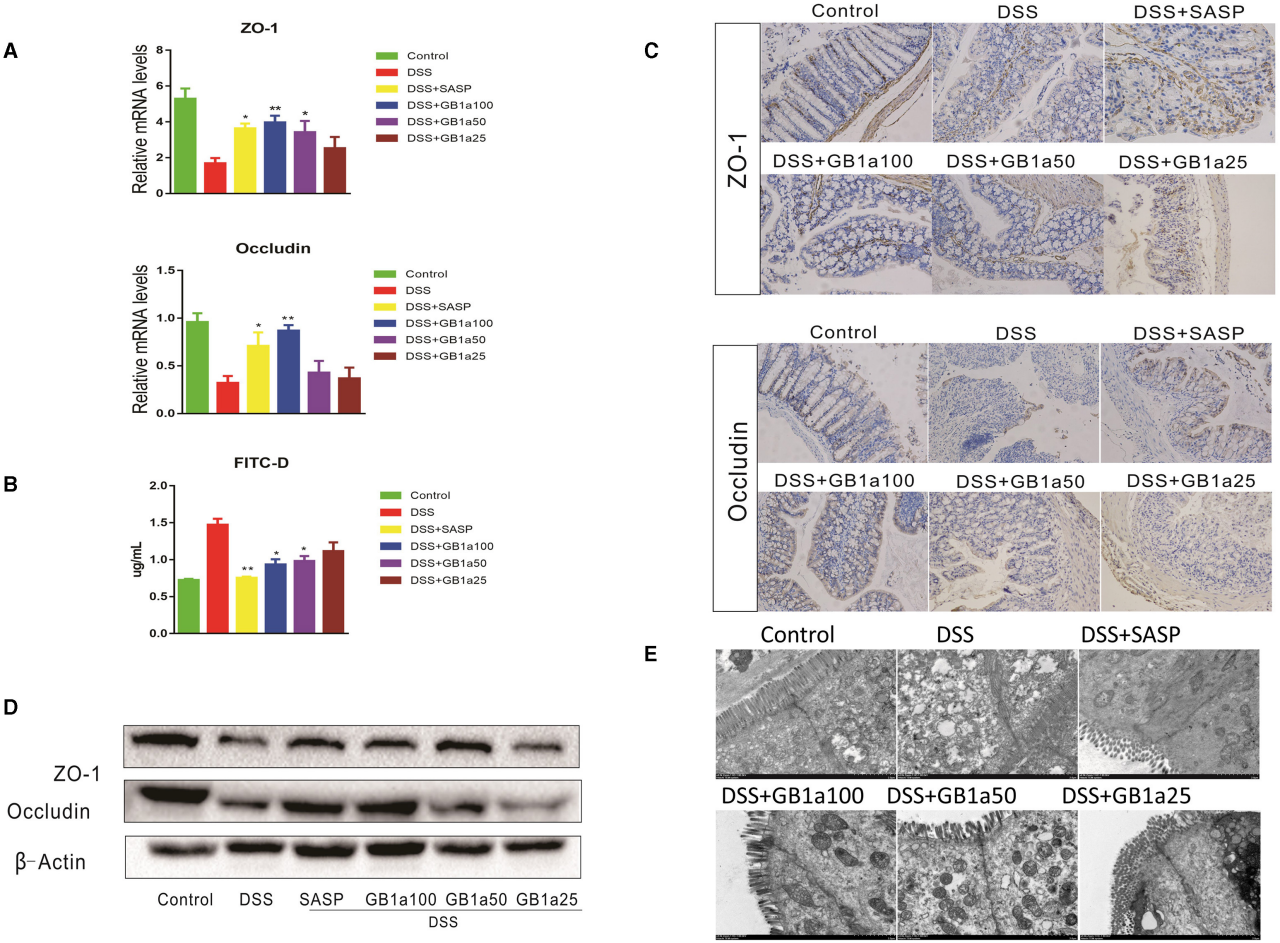
The protective effect of GB1a on the intestinal mucosa **(A)** GB1a treatment upregulated the expression of genes ZO-1 and Occludin. **(B)** GB1a administration reduced the levels of fluorescein isothiocyanate (FITC)-dextran in the serum of UC mice and improved mucosal permeability in the colon. **(C)** Representative images of immunohistochemical staining of ZO-1 and Occludin and Claudin2 in mouse colon sections. **(D,E)** Ultrastructural morphology of the intestinal mucosal barrier. Data are presented as means ± SD (*n* = 7/group). *p < 0.05 and **p < 0.01. vs the DSS-incubated group.

Damage to the intestinal mucosal barrier is an important cause of UC. We hypothesized that GB1a might have regulatory effects on DSS-induced tight junctions (TJ) molecules. Our results demonstrated that GB1a treatment increased the expression of ZO-1 and Occludin at the mRNA and protein levels (Figures 6A,D) and improved mucosal permeability and decreased the level of FITC in serum (Figure 6B). Immunohistochemistry staining results showed that GB1a treatment significantly enhanced ZO-1 and Occludin protein expression in inflamed colons compared to mice treated with DSS alone (Figure 6C) and were further verified by western blotting analysis (Figure 6D). Transmission Electron Microscope (TEM) revealed that GB1a (100 mg·kg^−1^) ameliorated DSS-induced loosening of the epithelial tight junction (TJ), increased colon space, caused the loss of microvilli, decreased desmosome density and decreased mitochondrial swelling. These changes acted to improve the integrity of the intestinal barrier (Figure 6E). In summary, our data indicated that GB1a treatment significantly repaired the damage of the intestinal mucosa by DSS-induced.

## Discussion

In this study, we showed that GB1a inhibited oxidative stress damage by activating the NRF2 pathway. GB1a regulated the balance between pro- and anti-inflammatory cytokines and maintained intestinal homeostasis by inhibiting activation of the NF-κB pathway. Also, GB1a reduced the permeability of the intestinal mucosa by repairing damage to the intestinal mucosal barrier and prevented endotoxins and bacteria from entering the blood circulation, ultimately relieving damage from the abnormal immune response.

Inflammatory bowel disease (IBD) includes UC and Crohn's disease (CD) (37). UC, also known as non-specific UC, is a chronic inflammatory disease where the main lesions occur in the colon. In 1859, the symptoms of UC were first described by Samuel Wilks (38) and included abdominal pain, diarrhea and bloody purulent stools. These symptoms are often accompanied by associated damage in the lymph nodes, skin, eyes, liver, and gallbladder (39).

Currently, the pathogenesis of UC is not fully understood. The process is thought to mainly involve genetic susceptibility, defects in the epithelial barrier, immune disorders and other environmental factors. Immune dysregulation is a key factor the affects the progression of the disease. Also, injury to the epithelial barrier injury is important in the pathogenesis of UC as pathogenic microorganisms and toxins can exacerbate ulcers by invading the intestinal tract.

Modern clinical approaches lack targeted drugs for the treatment of UC. Clinically, UC is mainly treated using strategies to regulate immune function, reduce intestinal mucosal edema and inhibit the production of inflammatory mediators. The main classes of drugs that are used to perform these functions are amino salicylic acid (5-ASA), adrenocorticosteroids, immunosuppressants, and inhibitors of inflammatory mediators. Whilst these drugs are effective, they have several side effects that impact the quality of life of patients.

Treatment with 5-ASA as a first-line therapy for UC may cause male infertility and folic acid deficiency. Also, various biological agents that target specific immune pathways have become recognized as potential treatments for UC (40, 41). Glucocorticoids are the most effective drugs used to inhibit acute active inflammation, yet their long-term use leads to hormone dependence and drug resistance (42). Immunosuppressive agents have more significant adverse reactions such as severe diarrhea, bone marrow suppression, hepatotoxicity and pancreatitis (43). Biological agents may be used to control the early stages of UC, however, these may result in adverse reactions including delayed allergic reactions, increased risk of infections and increased incidence of tumors (44). Therefore, there is an urgent need to develop more effective and safer alternative drugs in the treatment of UC.

Recently, researchers have identified natural compounds that are effective in the prevention and treatment of UC and other inflammatory diseases (45). These compounds are relatively non-toxic and have fewer side effects compared to established drugs. Natural products may have a high potential to improve the quality of life for patients with UC and also reduce the risk of cancer. Garcinia Kola Heckel is a flowering plant that produces a natural product with known anti-inflammatory, antioxidant, antiviral, antiulcer, and anti-bacterial activities (46–48). The crude extract of Garcinia kola is known to have protective effects against acetic acid-induced UC in rats. Kolaviron, a diflavonoid compound extracted from Garcinia Kola, has been shown to improve DSS-induced UC in rats through anti-inflammatory and antioxidant effects (49). In the current study, the biflavonoid compound, GB1a, was extracted from the seeds of Garcinia Kola (26, 27). GB1a is one of the most important active ingredients found in Garcinia Kola and has been reported to have analgesic, anti-inflammatory, antimalarial and antioxidant activities (50–52), however, it has not been evaluated in the treatment of UC.

Although the exact pathogenesis of UC remains unclear, accumulating evidence indicates that anti-oxidative and inflammatory pathways play significant roles (53, 54). Natural compounds are involved in the inflammatory and immune responses of the UC intestine. The integrity and repair of the mucosal barrier in the colon are critically important in improving the symptoms of UC (45, 55) and developing effective treatments for UC (56).

A major feature of UC is the development of severe inflammation in response to impaired immune responses in which NF-κB signaling pathways play a central role (57, 58). The release of related inflammatory cytokines and pro-inflammatory mediators after activation of the NF-κB pathway plays a crucial role in UC. These changes include elevated levels of TNF-α, IL-6, and IL-1β along with decreased levels of anti-inflammatory cytokines such as IL-10 (8, 9). In the current study, natural compounds had pronounced inhibitory effects on the NF-κB pathway by reducing the expression of inflammatory factors and promoting the expression of anti-inflammatory factors that acted to improve the symptoms of UC.

Curcumin is a natural hydrophobic polyphenol that has a variety of pharmacological effects in UC (59). Curcumin has been shown to down-regulate the expression of pro-inflammatory cytokines (IL-1, IL-6, IL-8, and TNF-α) by regulating the NF-κB/IκB pathway and reduce inflammatory cell infiltration in several experimental models. Saikosaponin-d improves dextran sulfate sodium-induced colitis by inhibiting activation of NF-κB signaling and regulating the intestinal microbiota in mice. Also, nutmeg reduces TNF-α, IL-6, and IL-1β levels in LPS induced mouse serum by blocking nuclear translocation of endotoxin shock and inhibits binding in LPS stimulated macrophages (60).

Cardamonin is another natural compound that blocks the nuclear translocation of NF-κBp65 in a mouse model of endotoxin shock. Cardamonin can reduce the levels of TNF-α, IL-6, and IL-1β secretion in LPS-induced mouse blood serum and inhibits NF-κB DNA-binding in LPS-stimulated macrophage cells (61). Our results revealed that GB1a administration decreased the expression of TNF-α, IL-6 mRNA and repressed NF-κBp65 protein expression and nuclear translocation by inhibiting activation of the NF-κB pathway. These data demonstrate that GB1a can effectively reduce inflammatory damage and highlight the potential for the therapeutic application of GB1a in the treatment of UC.

Neutrophil infiltration, free radical formation and increased oxidative stress are known biological mechanisms of UC (62, 63). Oxidative stress plays a key role in the development of many diseases and is usually accompanied by the production of a large amounts of oxygen free radicals. This directly causes oxidative damage to macromolecules such as DNA, proteins and lipids, destroying cell membranes, and other cellular structures. By stimulating the expression of cytokines and adhesion molecules, oxidative damage mediates inflammation, and the immune response to enhance tissue damage. The presence of oxygen free radicals can also indirectly activate apoptotic signaling pathways through the inhibition of mitochondrial function (64). During the development of UC, the oxidative burst of infiltrating macrophages leads to the production of large amounts of reactive oxygen species in the inflamed tissues of patients. This oxidative burst leads to the destruction of colon tissue and decreases epithelial permeability causing intestinal inflammation (13).

The nuclear factor erythroid 2-related factor 2 (Nrf2) signaling pathway is a defense system that regulates the expression of antioxidant proteins and the transcription of genes encoding detoxification enzymes. *In vivo* studies have shown that the Nrf2 signaling pathway also plays an important role in the improvement of UC (65–67). In our study, for the first time, we report that GB1a supplementation can effectively improve mitochondrial and oxidative stresses by reducing ROS in a Nrf2 dependent manner suggesting a strong link between Nrf2 and oxidative stress during the progression of UC.

The persistent inflammatory response in patients with UC compromises the integrity of the colonic mucosa through sustained cytokine release (9). The mechanical barrier of the intestinal mucosa is particularly important in the treatment of UC. The main structure of the mechanical barrier is formed by tight junction proteins (TJs) that are composed of claudin, zos and connexins (68). Also, the intestinal mucosal barrier plays a vital role in maintaining the barrier function to protect against intestinal allergens, toxins, and pathogens (59).

During the development of UC, the destruction of the intestinal mucosal barrier activates intestinal inflammation to promote the development of colon cancer (69). Previously, it has been shown that changes in the composition of colon mucus in UC promotes damage to the colon mucosal barrier. This leads to immune activation of symbiotic microbial communities and promotes the progression of UC diseases (70, 71). The results presented in this study demonstrate that GB1a can effectively increase the expression of the tight junction protein ZO-1 and Occludin in UC mice. The serum FITC content of the UC mice decreased after GB1a treatment which effectively alleviated the permeability of the colon mucosa toward maintaining the normal physiological function of the colon mucosa.

In summary, this is the first study to demonstrate the protective effects of GB1a on DSS-induced mouse UC. The underpinning molecular mechanisms of GB1a are potentially associated with the activation of Nrf2, protection of intestinal mucosa and the inhibition of NF-κB-mediated proinflammatory signaling.

## Data Availability Statement

The original contributions presented in the study are included in the article/Supplementary Material, further inquiries can be directed to the corresponding author/s.

## Ethics Statement

The animal study was reviewed and approved by Animal Ethics Committee of Guangzhou University of Traditional Chinese Medicine.

## Author Contributions

YY, CZ, XL, CD, and WG performed the experiments and data analysis and wrote the manuscript. CL and QWu contributed to the study design and acquisition and analysis of data. QWa, QX, and XH contributed to the drafting of the manuscript. JS designed the experiments, provided funding support, and performed a critical revision of the manuscript. All authors contributed to the article and approved the submitted version.

## Funding

This work was supported by the National Natural Science Foundation of China [Grant Numbers 81873218 and 81773969], the National Major Science and Technology Projects of China [Grant Number 2018ZX09303008], the Project of Traditional Chinese Medicine Bureau of Guangdong [Grant Number 2019 (43)], the YangFan Innovative And Entrepreneurial Research Team Project [Grant Number 2014YT02S008], and Science and Technology Program of Guangzhou [Grant Number 202002020032].

## Conflict of Interest

The authors declare that the research was conducted in the absence of any commercial or financial relationships that could be construed as a potential conflict of interest.

## Publisher's Note

All claims expressed in this article are solely those of the authors and do not necessarily represent those of their affiliated organizations, or those of the publisher, the editors and the reviewers. Any product that may be evaluated in this article, or claim that may be made by its manufacturer, is not guaranteed or endorsed by the publisher.
