# Supplementary material for: GB1a Ameliorates Ulcerative Colitis via Regulation of the NF-κB and Nrf2 Signaling Pathways in an Experimental Model
Source: Front Med (Lausanne). 2021 Sep 7;8:654867. doi: 10.3389/fmed.2021.654867 (PMC8452853; doi:10.3389/fmed.2021.654867)

**Methods**

**Transfection of small interfering RNA (siRNA) .**

HCoEpic cells were seeded in 6-well plates for 24 h. The pre-designed siRNA (NO.stB0003575c) and control disrupted siRNA (NO.siN0000001-1-5) were purchased from RiboBio (Guangzhou, China). Then, The siRNA transfection complex was then prepared in accordance with the protocol of the riboFECT™CP transfection kit(RiboBio; Cat#C10511). Dilute the NF-κBp65 siRNA with riboFECT™ CP buffer to a final concentration of 50 nM, and then add an appropriate amount of riboFECT CP transfection reagent to the siRNA solution and mix well. The transfection mixture was prepared by incubation at room temperature for 10 min. Then add each group of transfection compounds to the cell culture medium of the corresponding wells and mix gently. After 6 h of incubation, fresh medium was added and incubated for another 24 h. Cells were treated with 40μM GB1a for 24 h and collected for and RT-PCR.

**Molecular modeling and docking studies.** All calculations were performed using SYBYL-X 2.2.1 software and the results were analyzed the Discover Studio 2016 software. The GB1a structure was downloaded from the PubChem database and optimized to obtain the most stable structure using the Minimize molecular mechanics program. The three-dimensional crystal structures of the NF-κB and Nrf2 proteins were downloaded from the RCSB PDB database. Structural modifications of the proteins such as hydrogenation, repair of side chains, charging and extraction of embedded ligands were identified. The Total-Score and C-Score values were cross-referenced and used to evaluate the molecular docking effect. The molecular surface properties were calculated by MOLCAD and the binding modes and mechanisms of interaction of the proteins and small molecules were analyzed.

**Immunohistochemistry analysis.** Mouse colon tissue samples were fixed in 4% paraformaldehyde for 24 h before being dehydrated and embedded in paraffin. Standard immunohistochemistry staining was performed. Tissue sections were incubated with the primary antibodies (ZO-1 and Occludin antibody) at 4°C overnight followed by incubation with a streptavidin-biotin-peroxidase conjugated secondary antibody for 30 min at room temperature. For the negative control, the primary antibody was replaced with 1% nonimmune serum in PBS. The expression of ZO-1 and Occludin was scored by measuring the integrated optical densities of at least three fields of view per slide using Image Pro-plus 6.0 software (Media Cybernetics, Rockville, VA, USA).

**Immunofluorescence staining****.** HCoEpic cells were seeded on glass coverslips and incubated overnight. Cells were incubated with TNF-α (30 ng·ml^-1^) and TNF-α plus 20 μM or 40 μM GB1a (TNF-α+GB1a group) for 24 h. Cells were then fixed with 4% paraformaldehyde for 20 min at room temperature. After washing with PBS, cells were permeabilized with 0.1% Triton X-100 in PBS for 10 min at room temperature. The colon tissues were fixed in 4% paraformaldehyde for 24 h before being dehydrated and embedded in paraffin. Standard immunofluorescence procedures were performed. After incubation with blocking buffer 5% BSA for 30 min, cells or tissues were incubated with rabbit anti-NF-κB p65 antibody (1:100 dilution) or anti-Nrf2 antibody (1:100 dilution) at 4 °C overnight. The samples were then incubated with CoraLite488-conjugated secondary antibody (1:500) for 1 h at room temperature. The glass coverslips were washed with PBS solution and mounted using mounting medium (with DAPI) (Solarbio) was added. Fluorescence images were obtained using a confocal laser scanning microscope (LEICA, Wetzlar, Germany).

**RNA isolation and quantitative RT-PCR (qRT-PCR).** Total RNA was extracted from the samples using TRIzol reagent. The quality and quantity of the total RNA were determined using a NanoDrop 2000 Spectrophotometer (Thermo Fisher Scientific, Waltham, MA, USA). Reverse transcription was performed using 5X All-In-One RT Master Mix (Applied Biological Materials Inc). qPCR was performed using PowerUp™ SYBR™ Green Master Mix (Thermo Fisher Scientific) on a QuantStudio™ 6 Flex Real-Time PCR System (Thermo Fisher Scientific). The specific primer sequences are presented in Supplementary Table 2.

**Supplementary Tables**

Table 1. GB1a NMR Spectroscopy data（in DMSO-d_6_）

| NO | Compound A | | Compound B | |
| --- | --- | --- | --- | --- |
|  | δ H | δ C | δ H | δ C |
| I-C-3 | 4.67 (d,12.1)  4.53 (d,12.2) | 47.37 | 4.67 (d,12.1)  4.53 (d,12.2) | 47.7 |
| II-C-3 | 2.59(d,15.7)  2.75 (d,19.8) | 43.15,42.70 | 2.59 (d,15.7)  2.75 (d,19.8) | 43.0 |
| I-C-2 | 5.69 (d,12.1)  5.56 (m)  5.38 (m) | 81.77,81.29 | 5.69 (d, 12.1 )  5.56 (m)  5.38 (m) | 81.4 |
| II-C-2 | 4.67 (d,12.1) | 78.41,78.22 | 4.67 (d,12.1) | 78.3 |
| I-A-6 | 5.91 (d,8.9)  5.94 (s) | 96.10,95.53 | 5.91 (d,8.9)  5.94 (s) | 96.0 |
| I-A-8 | 5.82 (s) | 95.13 | 5.82 (s) | 95.0 |
| II-A-6 | 5.87 (s)  5.78 (s) | 95.01 | 5.87 (s)  5.78 (s) | 94.9 |
| II-A-8 |  | 101.62,101.41 |  | 101.3 |
| I-A-4a |  | 101.31 |  | 101.3 |
| II-A-4a |  | 101.14,101.05 |  | 101.0 |
| I-B-3' | 6.64 (d,7.2) | 114.68,114.76 | 6.64 (d,7.2) | 114.5 |
| I-B-5' | 6.64 (d,7.2) | 114.68,114.76 | 6.64 (d,7.2) | 114.5 |
| II-B-2' | 7.14 (d,8.2)  7.22 (d,8.1) | 126.68 | 7.14 (d,8.2)  7.22 (d,8.2) | 127.3 |
| II-B-5' | 6.85 (d,8.1)  6.71 (d,8.0) | 115,115.24 | 6.85 (d,8.1)  6.71 (d,8.0) | 114.9 |
| II-B-6' | 7.14 (d,8.2)  7.22 (d,8.1) | 126.68 | 7.14 (d,8.2)  7.22 (d,8.1) | 127.3 |
| I-B-1' |  | 128.07,127.99 |  | 127.9 |
| Il-B-1' |  | 129.45,129.13 |  | 128.9 |
| I-B-2' | 7.12 (d,7.7)  6.85 (d,8.1) | 127.81 | 7.12 (d,7.7)  6.85 (d,8.1) | 128.5 |
| 1-B-6' | 7.12 (d,7.7)  6.85 (d,8.1) | 128.90,128.98 | 7.12 (d,7.7)  6.85 (d,8.1) | 128.5 |
| II-B-3' | 6.85 (d,8.1)  6.71 (d,8.0) | 115,115.24 | 6.85 (d,8.1)  6.71 (d,8.0) | 114.9 |
| II-B-4' |  | 157.72,157.54 |  | 157.1 |
| I-B-4' |  | 157.15 |  | 157.1 |
| I-A-5 |  | 163.85,163.63 |  | 163.4 |
| I-A-7 |  | 166.38 |  | 165.9 |
| I-A-8a |  | 162.79,162.65 |  | 162.3 |
| 11-A-5 |  | 162.47,162.02 |  | 162.3 |
| II-A-7 |  | 164.97,164.49 |  | 164.3 |
| II-A-8a |  | 159.76,160.71 |  | 162.0 |
| I-C-4 |  | 196.11 |  | 195.2 |
| II-C-4 |  | 196.81,196.58 |  | 196.1 |

A Spectrogram data; B Documentation data

Table 2. Primer sequences for gene amplification

| Primer | Sequences | |
| --- | --- | --- |
| TNF-a(h) | | F1:CCTCTCTCTAATCAGCCCTCTG |
|  | | R1:GAGGACCTGGGAGTAGATGAG |
| IL-1B(h) | | F1:ATGATGGCTTATTACAGTGGCAA |
|  | | R1:GTCGGAGATTCGTAGCTGGA |
| IL-6(h) | | F1:ACTCACCTCTTCAGAACGAATTG |
|  | | R1:CCATCTTTGGAAGGTTCAGGTTG |
| Nrf2(h) | | F1:TCAGCGACGGAAAGAGTATGA |
|  | | R1:CCACTGGTTTCTGACTGGATGT |
| HO-1(h) | | F1:AAGACTGCGTTCCTGCTCAAC |
|  | | R1:AAAGCCCTACAGCAACTGTCG |
| COXII(h) | | F1:CTGGAAGCCAAGCACTTTTGG |
|  | | R1:AGTCCGGAGCGGGAAGAAC |
| NF-κB p65(h) | | F1:ATGTGGAGATCATTGAGCAGC |
|  | | R1:CCTGGTCCTGTGTAGCCATT |
| GAPDH(h) | | F1:GGAGCGAGATCCCTCCAAAAT |
|  | | R1:GGCTGTTGTCATACTTCTCATGG |
| TNF-a(m) | | F1:CAGGCGGTGCCTATGTCTC |
|  | | R1:CGATCACCCCGAAGTTCAGTAG |
| IL-6(m) | | F1:TAGTCCTTCCTACCCCAATTTCC |
|  | | R1:TTGGTCCTTAGCCACTCCTTC |
| Nrf2(m) | | F1:CTTTAGTCAGCGACAGAAGGAC |
|  | | R1:AGGCATCTTGTTTGGGAATGTG |
| HO-1(m) | | F1:AGGTACACATCCAAGCCGAGA |
|  | | R1:CATCACCAGCTTAAAGCCTTCT |
| xMarfK(m) | | F1:ATGACGACTAATCCCAAGCCC |
|  | | R1:CATCATCGCTAAGAACAGGGG |
| ZO-1(m) | | F1:GCCGCTAAGAGCACAGCAA |
|  | | R1:GCCCTCCTTTTAACACATCAGA |
| Occludin(m) | | F1:TGAAAGTCCACCTCCTTACAGA |
|  | | R1:CCGGATAAAAAGAGTACGCTGG |
| CCl20(m) | | F1:ACTGTTGCCTCTCGTACATACA |
|  | | R1:GAGGAGGTTCACAGCCCTTTT |
| CXCL1(m) | | F1:ACTGCACCCAAACCGAAGTC |
|  | | R1:TGGGGACACCTTTTAGCATCTT |
| CCL5(m) | | F1:TTTGCCTACCTCTCCCTCG |
|  | | R1:CGACTGCAAGATTGGAGCACT |

**Supplementary Figures**

**Figure legends**

**Figure 1.**

1. The HPLC profile of GB1 extracted from Garcinia Kola nuts was analyzed at 360 nm. The sample was 99.7% pure.
2. Structural information of the monomer compounds obtained by nuclear magnetic resonance (NMR) analysis.
3. The structure of GB1a, a biflavonoid compound extracted from Garcinia Kola nuts.

**Figure 2.**

The mRNA expression of NF-κBp65, TNF-α, IL-6 and IL-1β after treatment to NF-κBp65 siRNA and GB1a Data are presented as means±SD (n=5/group). ***p<0.001.vs the control group.

**Supplementary Figure 1**


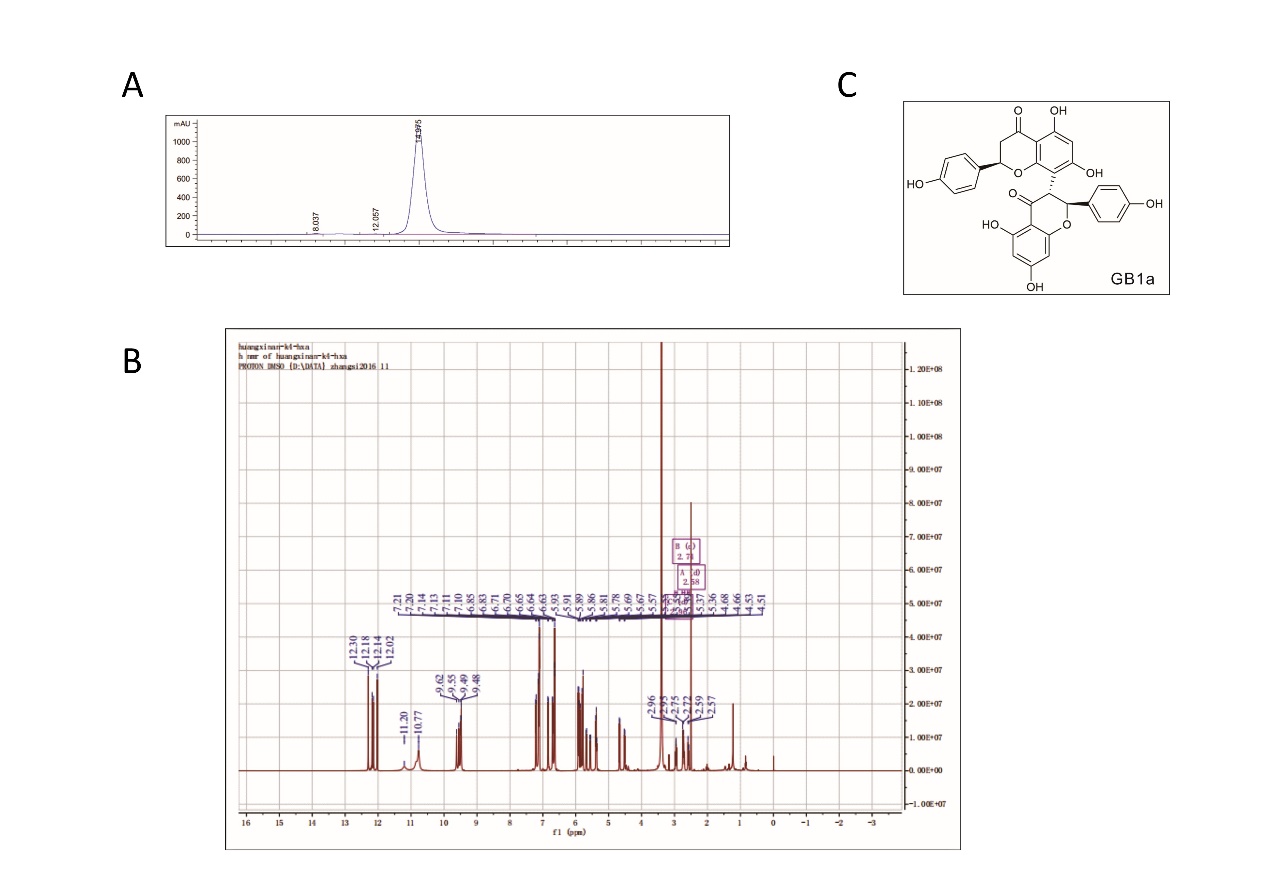


**Supplementary Figure 2**


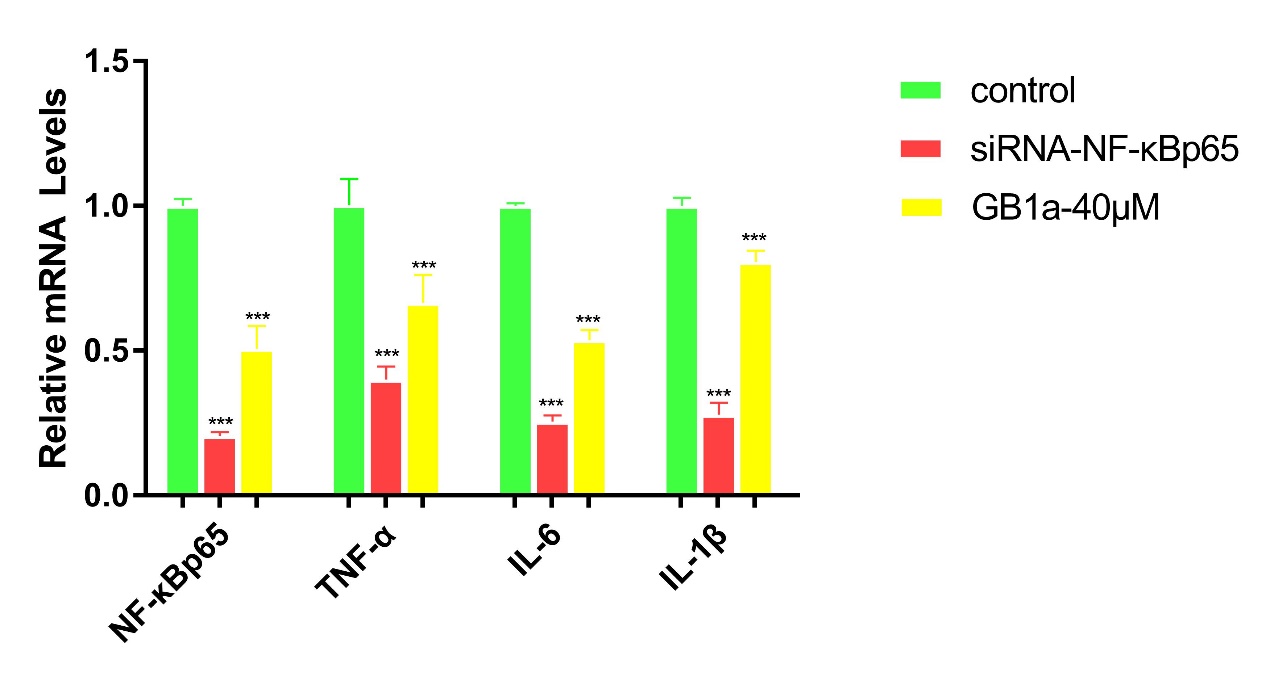

Supplement: Supplementary file 1 [file Data_Sheet_1.docx]
